# Lack of XPC leads to a shift between respiratory complexes I and II but sensitizes cells to mitochondrial stress

**DOI:** 10.1038/s41598-017-00130-x

**Published:** 2017-03-13

**Authors:** Mateus P. Mori, Rute A. P. Costa, Daniela T. Soltys, Thiago de S. Freire, Franco A. Rossato, Ignácio Amigo, Alicia J. Kowaltowski, Aníbal E. Vercesi, Nadja C. de Souza-Pinto

**Affiliations:** 1 Departamento de Bioquímica, Instituto de Química, Universidade de São Paulo (USP), São Paulo, SP Brazil; 2 Department of Clinical Pathology, School of Medical Sciences, Universidade Estadual de Campinas (UNICAMP), Campinas, SP Brazil

## Abstract

Genomic instability drives tumorigenesis and DNA repair defects are associated with elevated cancer. Metabolic alterations are also observed during tumorigenesis, although a causal relationship between these has not been clearly established. Xeroderma pigmentosum (XP) is a DNA repair disease characterized by early cancer. Cells with reduced expression of the XPC protein display a metabolic shift from OXPHOS to glycolysis, which was linked to accumulation of nuclear DNA damage and oxidants generation via NOX-1. Using XP-C cells, we show that mitochondrial respiratory complex I (CI) is impaired in the absence of XPC, while complex II (CII) is upregulated in XP-C cells. The CI/CII metabolic shift was dependent on XPC, as XPC complementation reverted the phenotype. We demonstrate that mitochondria are the primary source of H_2_O_2_ and glutathione peroxidase activity is compromised. Moreover, mtDNA is irreversibly damaged and accumulates deletions. XP-C cells were more sensitive to the mitochondrial inhibitor antimycin A, an effect also prevented in XPC-corrected cells. Our results show that XPC deficiency leads to alterations in mitochondrial redox balance with a CI/CII shift as a possible adaptation to lower CI activity, but at the cost of sensitizing XP-C cells to mitochondrial oxidative stress.

## Introduction

Although it is well known that tumor formation depends on a multitude of molecular events, mutation accumulation is a *bona fide* basis for cellular transformation^1^. The direct relationship between genomic instability and cancer can be best appreciated in inherited diseases that predispose affected individuals to early emergence of neoplasia.

Mutations in genes that encode for DNA repair proteins cause cancer-prone syndromes^2^. DNA repair diseases usually lead to onset of cancer within the first two decades of the patient’s life. Xeroderma pigmentosum (XP) is one of these inherited diseases, characterized by photosensitivity, hyperpigmentation, premature skin aging and a 10,000-fold increase in the incidence of skin malignancies^3^. Mutations in eight genes have been described to give rise to XP: XP-A to XP-G and a variant form, XP-V (*POLH*) that encodes for an error-prone translesion synthesis DNA polymerase^4^. All seven canonical XP proteins, from XPA to XPG, are involved in nucleotide excision repair (NER) – for an extensive review on NER see ref. 5.

Although the elevated cancer predisposition in XP patients could be directly explained by the DNA repair defect, a more severe set of phenotypes related to a vast degeneration in the central nervous system raised questions about other functions of XP proteins. Some XP patients also present Cockayne syndrome (CS) features, including growth and developmental retardation, neurodegeneration and premature aging. Some mutations in *XPD* and *XPG* can give rise to a combined XP/CS phenotype, while mutations in *CSA* and *CSB*, two components of the transcription coupled NER sub-pathway (TCR), give rise only to CS^6^. It has been speculated that TCR deficiency was the molecular cause of the neurological phenotype, but this hypothesis was partially discredited by evidence that some individuals affected by UV-sensitive syndrome (UVSS) carried mutations in *CSA* and *CSB* genes without any discernible neurodegeneration^7, 8^. Thus, some authors argued that the neurodegeneration phenotype could be due to accumulation of oxidized damage, since cells from XP-G (with a XP/CS phenotype), CS-A and CS-B patients were sensitive to oxidative stress^9^. Nonetheless, cells from XP-C patients also show increased sensitivity to oxidants while these patients do not manifest neurological abnormalities^10, 11^. In the global genome NER sub-pathway (GGR), the XPC protein participates in the initial step of lesion recognition in association with its binding partners hRAD23B and centrin-2^6^. Although oxidatively-induced DNA damage is repaired primarily by the BER pathway, a role for XPC in the repair of oxidized DNA lesions has been demonstrated. XP-C cells accumulate 8-oxoGua in nuclear DNA after treatment with oxidizing agents, and the XPC protein interacts physically and functionally with OGG1, stimulating its catalytic activity^10^.

There is growing evidence that DNA repair defects lead to mitochondrial dysfunction. Mitochondrial dysfunction has been well documented in CS, as CS-A and CS-B cells show impaired mitochondrial DNA (mtDNA) repair^9, 12, 13^, redox imbalance^14^ and increased mitochondrial autophagy^15^. Likewise, in cells from ataxia telangectasia (AT) patients, with a mutated ATM protein, as well as in ATM knockout mice, mitochondrial bioenergetics^16, 17^ and mtDNA repair defects^18^ have also been demonstrated. CSA, CSB and ATM proteins have been localized in mitochondria, and a direct role for these in mtDNA stability has been demonstrated^12, 13, 16^. However, not all DNA repair disorders with neurodegeneration can be directly linked to mtDNA repair. De Sanctis-Cacchione patients bearing mutation in *XPA* gene manifest late neurological symptoms that has been linked to dysfunctional mitophagy. Since XPA is a downstream effector of DNA damage recognition in both GGR and TCR, incomplete DNA repair events keep PARP1 activated, depleting NAD^+^ and altering NADH/NAD^+^ ratio. Nutrient-sensitive SIRT1 also uses NAD^+^ to deacetylate target proteins, including transcription factors that stimulate expression of PGC-1α, a master mitochondrial biogenesis regulator, which, therefore, is also downregulated. Because PGC-1α regulates UCP2 expression, mitochondria from XP-A cells show increased mitochondrial membrane potential leading to elevated ROS generation, due to blocked electron flow with increased reverse electron flow, and to decreased mitophagy^19^. In line with these findings, it is well known that mitochondrial dysfunction is also a common feature of aging and age-associated diseases, such as cancer and neurodegeneration^20^, conditions that have been causally linked to genomic instability^21^.

Mitochondrial dysfunction was also demonstrated in human keratinocytes after XPC knockdown^22, 23^. These effects were linked to nuclear DNA damage accumulation and NOX-induced hydrogen peroxide generation, but no direct effect of XPC knockdown on mitochondrial bioenergetics was investigated. Thus, we investigated whether XPC is directly involved in mitochondrial function and mtDNA maintenance. Using transformed and primary fibroblasts from XP-C patients, we evaluated mitochondrial bioenergetic parameters, ROS production, mtDNA integrity and cytotoxicity of mitochondrial inhibitors. We report that lack of XPC leads to impaired complex I (CI – NADH dehydrogenase complex) activity and altered mitochondrial H_2_O_2_ production/clearance, with consequent mtDNA damage accumulation. XP-C cells overcome complex I dysfunction upregulating complex II (CII – succinate dehydrogenase complex) components, but at a cost of increased cellular sensitivity to mitochondrial stressors. These results suggest that absence of XPC impacts mitochondrial maintenance and that mitochondrial dysfunction may contribute to tumor formation in an XPC-deficient background.

## Results

### Complex I supported mitochondrial respiration is impaired in XP-C

**Figure 1:**
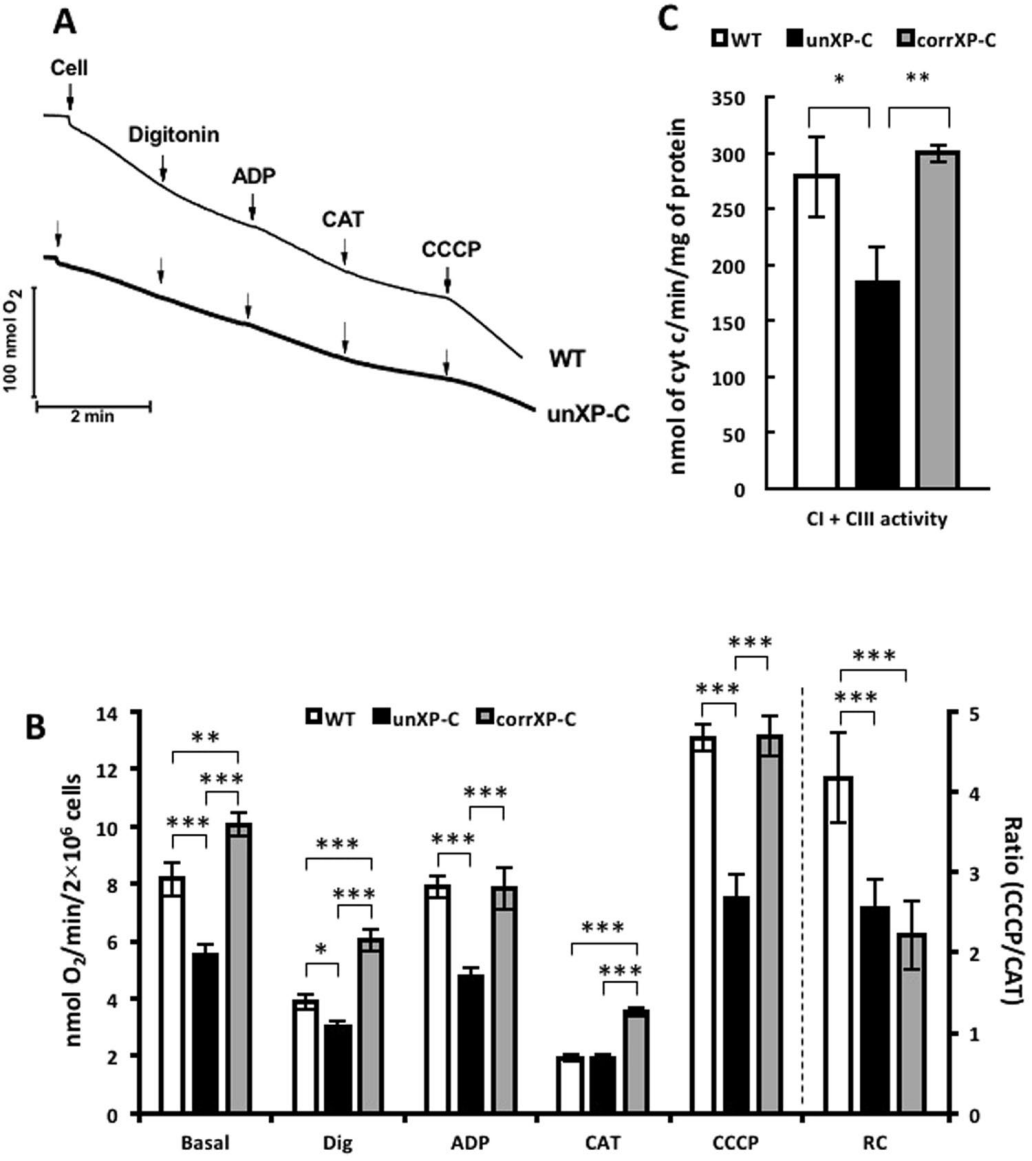
Complex I-supported mitochondrial function is impaired in XP-C cells. (**A**) O_2_ consumption for MRC-5 (WT), XP4PA (unXP-C) and XP4PA expressing XPC (corrXP-C) was measured as described, using 4 × 10^6^ cells in standard reaction medium containing 5 mM CI substrate, with the subsequent additions of 30 µM Digitonin, 100 µM ADP, 4 µM CAT and 1 µM CCCP. The figure shows a representative trace OCR experiment. (**B**) The rates of O_2_ consumption and respiratory control (RC, ratio between CCCP/CAT) were calculated from data obtained in the experiments represented in (**A**). (**C)** CI + CIII activity was measured using 5 µg of crude mitochondrial protein. Reduction of cyt c was monitored at 550 nm at 37 °C for 5 min. For the calculations, only the region where the slopes of the reactions were linear were considered. The data represent mean ± SD of 4 independent experiments. *p < 0.05, **p < 0.01 and ***p < 0.001.

Recently, Rezvani and colleagues^22, 23^ showed that XPC silencing in keratinocytes leads to a NOX-dependent metabolic shift from oxidative phosphorylation (OXPHOS) to glycolytic energy production. To directly investigate whether mitochondrial respiration is impaired in XP-C cells we measured oxygen consumption rates (OCR) of the different mitochondrial electron transport chain (ETC) complexes in transformed fibroblasts from normal and XP-C individuals (WT and unXP-C, respectively; see Suppl. Fig. S1 for XPC expression levels). In non-permeabilized cells, respiring only on endogenous substrates, OCR was already significantly lower in unXP-C cells, showing a 40% decrease when compared with WT (Fig. 1A and B, basal). After permeabilizing the plasma membrane with digitonin, to give access to added CI substrates, unXP-C cells still showed a significantly lower OCR, indicating that endogenous substrate availability was not limiting (Fig. 1B, dig). Phosphorylating respiration, measured after ADP addition, was also significantly lower in unXP-C cells, again showing around 40% decrease, from 7.91 ± 0.39 nmol O_2_/min/2 × 10^6^ cells in WT to 4.73 ± 0.36 in unXP-C cells (Fig. 1B, ADP). Maximum, uncoupled respiration, measured after CCCP addition, was also significantly diminished in unXP-C cells, again, close to 40% lower (Fig. 1B, CCCP). Surprisingly, OCR was completely recovered in XPC-corrected (corrXP-C) cells, which showed OCR similar to WT levels under all the conditions evaluated here. *In locus* genetic correction of the XP4PA cell line was confirmed by RT-qPCR and Western blotting and we observed approximately 53% and 60% of XPC protein and mRNA levels, respectively, in comparison with MRC-5 cell line (Suppl. Fig. S1). CorrXP-C cells show similar OCR at basal levels and even an increased digitonin-permeabilized OCR (Fig. 1B, dig: WT = 3.89 ± 0.27 *vs.* corrXP-C = 6.03 ± 0.38). Respiratory rates under phosphorylating conditions were similar in WT and corrXP-C cells, as well as maximum uncoupled respiration. Together, these results suggest that XPC absence leads to decreased CI function or that XPC is necessary for its normal function. On the other hand, OCR in presence of carboxyatractyloside (Fig. 1B, CAT) or oligomycin (not shown) was similar in unXP-C and WT cells, indicating that proton leak-associated mitochondrial respiration was not affected by lack of XPC. However, in corrXP-C cells, OCR in presence of CAT was increased in comparison with both WT and unXP-C cells, an indicative of increased proton leak. Thus, respiratory control (RC), representing the ratio between phosphorylating and non-phosphorylating respiration, is significantly decreased in mitochondria from unXP-C cells. Even though CI-linked maximum respiratory capacity is reverted in corrXP-C cells, RC is also decreased compared to WT, likely reflecting the increased proton leak-associated respiration after XPC correction (Fig. 1B).

To further elucidate if CI-linked decreased OCR was due to decreased CI activity, we directly measured CI + CIII activity in crude mitochondrial fractions (CMF). CMF obtained from WT cells reduced 278.6 ± 35.2 nmoles of cytochrome *c*/min/mg of protein *vs.* 184.4 ± 31.5 from unXP-C cells, while XPC expression in corrXP-C cells restored CI + CIII activity to WT levels (Fig. 1C). Accordingly, unXP-C CI + CIII activity was 34 and 41% lower than WT and corrXP-C cells, respectively, a reduction very similar to that observed in the OCR experiments. However, mRNA levels of both mitochondrial- (*MT-ND1* and *MT-ND4L*) and nuclear-encoded (*NDUFS1*) CI subunits were unchanged in XP-C cells (Suppl. Fig. 2A). Similarly, protein levels of two CI nuclear-encoded subunits were either unchanged (NDUFS3) or upregulated (NDUFS4) in unXP-C cells compared to corrXP-C cells (Suppl. Fig. 2B). Taken together, these results indicate that unXP-C cells show decreased CI-linked OCR due to reduced CI activity, despite normal expression levels of some of its protein subunits.

### Impaired CI function in XP-C does not lead to mitochondrial respiratory dysfunction

**Figure 2:**
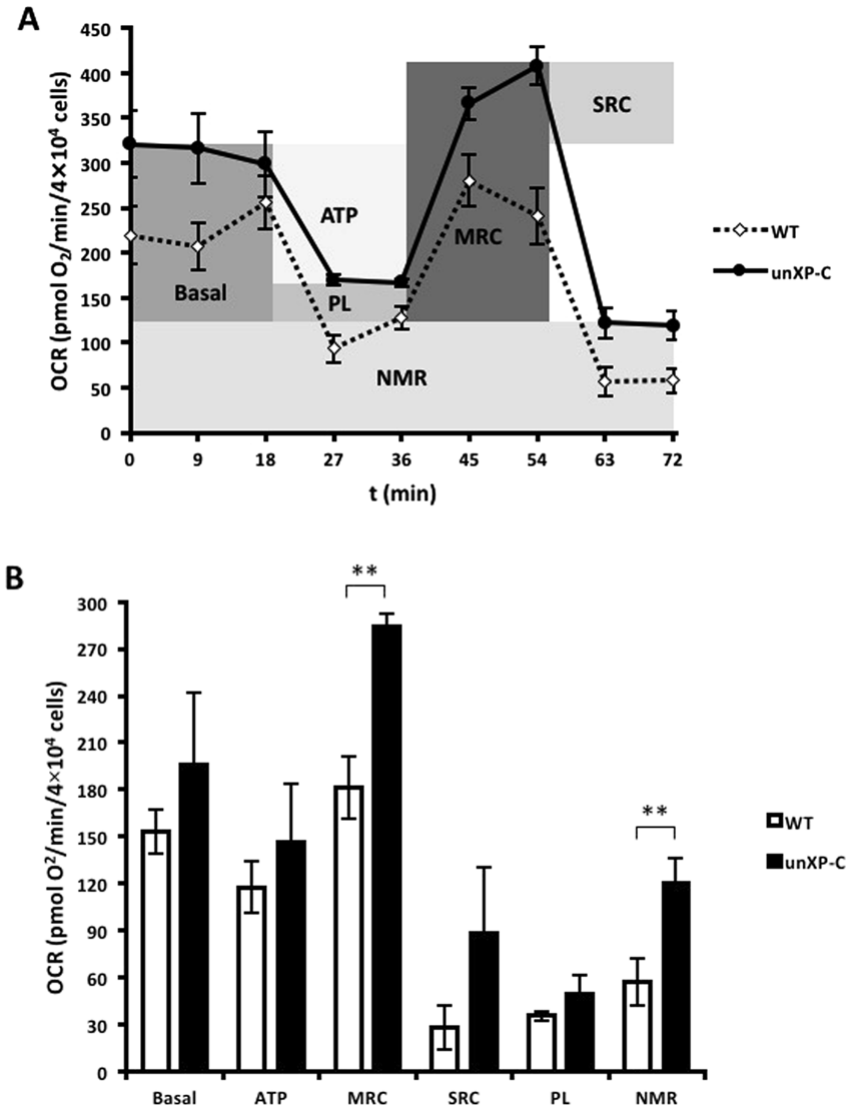
Oxygen consumption is not altered in intact XP-C fibroblasts. (**A**) O_2_ consumption was measured in MRC-5 (WT) and XP4PA (unXP-C) as described, using 4 × 10^4^ cells/well in XF Assay Medium containing 10 mM glucose, with the subsequent additions of 1 µM oligomycin (ATP synthase inhibitor), 200 nM CCCP (uncoupler) and 1 µM rotenone plus AA (CI and CIII inhibitors). The trace represents the mean ± SD of three independent experiments and a schematic illustration of respiratory parameters. (**B**) Calculated respiratory parameters: **Basal** (base line OCR minus rotenone plus AA OCR), **ATP** (ATP-linked OCR – base line OCR minus oligomycin OCR), **MRC** (maximum respiratory capacity – CCCP OCR minus rotenone plus AA OCR), **SRC** (spare respiratory capacity – CCCP PCR minus base line OCR), **PL** (proton leak – oligomycin OCR minus rotenone plus AA OCR), **NMR** (non-mitochondrial respiration – rotenone plus AA OCR). The data represent mean ± SD of 3 independent experiments. **p < 0.01**.**

OCR measurements in intact cells are especially informative because they reflect the use of all carbon sources of the cell, and ATP turnover (synthesis and degradation) includes every cellular reaction that demands ATP, directly or indirectly^24^. In order to evaluate the consequences of CI-impaired respiration on mitochondrial function in XP-C cells we performed OCR experiments in intact cells using the XF24 Extracelular Flux Analyzer (SeaHorse Bioscience). Although absolute OCR was increased in unXP-C compared to WT cells (Fig. 2A), when we corrected by discounting non-mitochondrial respiration, basal respiratory parameters were unchanged (Fig. 2B). Unexpectedly, unXP-C cells presented an increased maximum respiratory capacity compared to WT (*p* < 0.05), but this did not result in an increased spare respiratory capacity (Fig. 2B). UnXP-C cells also displayed increased non-mitochondrial respiration. This was not surprising since Rezvani and co-workers^22, 23^ already demonstrated increased NOX-1 activity in XPC silenced keratinocytes. Although XP-C cells present decreased NADH-linked respiration, the sum of energetic input/output in intact cells suggested that mitochondrial respiration is not altered in XP-C.

### Nuclear-encoded complex II activity is upregulated in XP-C cells

**Figure 3:**
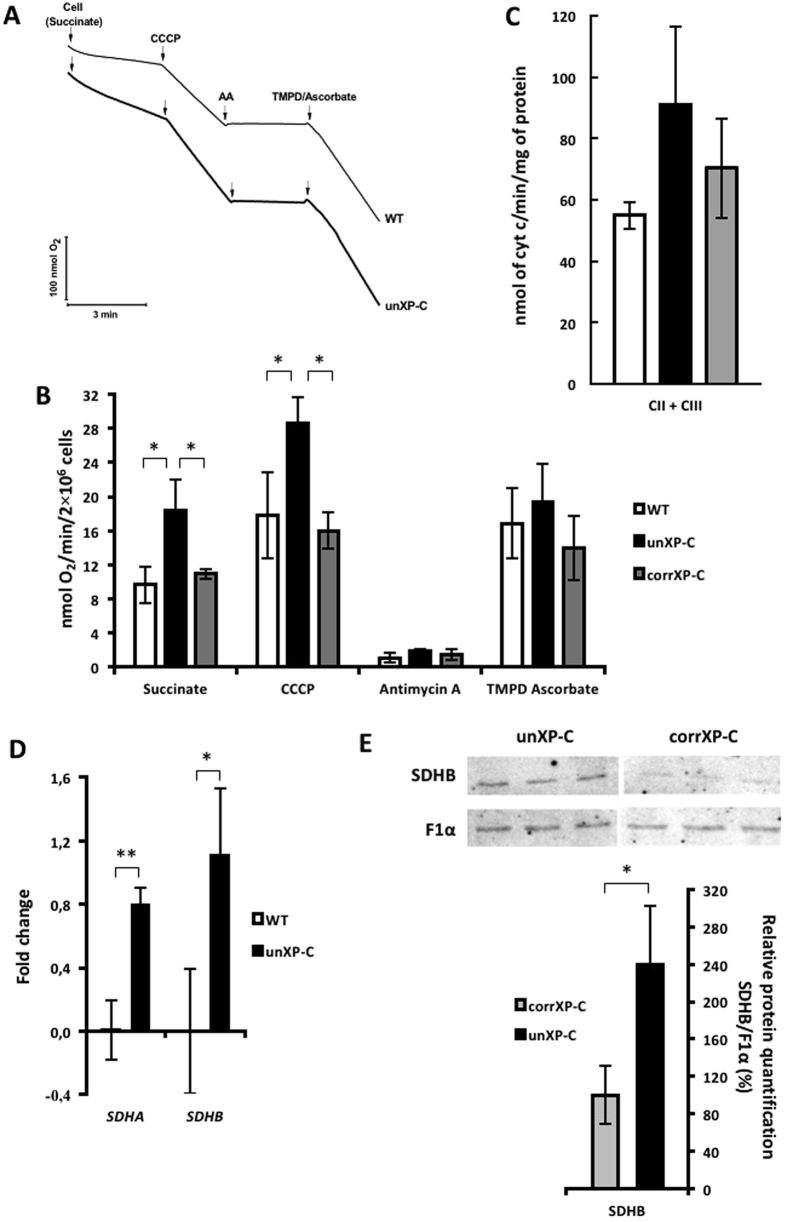
Respiratory complex II, but not complexes III and IV, is upregulated in XP-C cells. (**A**) OCR was measured using 4 × 10^6^ cells in standard reaction medium. Cells were permeabilized with 30 µM digitonin, with the following sequential additions: 5 mM succinate, 0.25 µM CCCP, 1 µM AA and 200 µM TMPD + 2 mM ascorbate. The figure shows a representative trace OCR experiment. (**B**) The rates of oxygen consumption were calculated from data obtained as shown in panel A. (**C**) CII + CIII activity was measured using 12 µg of crude mitochondrial protein. Reduction of cyt c was monitored at 550 nm at 37 °C for 5 min. It was only considered the range where slopes of the reactions were linear. (**D**) Expression analysis of *SDHA* and *SDHB* genes by RT-qPCR. Fold-change in mRNA expression of unXP-C relative to the WT cell line. ACTB was set as reference gene for all genes analyzed. (**E**) Western blotting of CI subunit SDHB and ATPase subunit F1α. SDHB protein expression level was normalized against F1α in unXP-C relative to corrXP-C cells. The data represent mean ± SD of 3 independent experiments. *p < 0.05 and **p < 0.01.

Since we demonstrated that CI activity was decreased in XP-C cells without significant mitochondrial respiratory impairment in intact cells, we measured OCR using CII-linked substrates to check whether CII could be compensating for impaired CI respiration. Mitochondria from XP-C cells energized with succinate, feeding electrons directly into CII, show a 1.7 fold higher OCR than WT mitochondria, from 9.65 ± 2.13 nmol O_2_/min/2 × 10^6^ cells in WT to 18.34 ± 3.59 in unXP-C cells (Fig. 3A and B). Likewise, uncoupled respiration supported by succinate was also significantly higher in the unXP-C cells. In accordance with the observation that XPC correction reverts XP-C-induced CI downregulation, CII activity also returns to WT levels in corrXP-C cells (WT = 9.65 ± 2.13 *vs.* corrXP-C = 10.95 ± 0.60). We also observed a trend toward an increase in CII + CIII activity in crude mitochondrial fractions from unXP-C compared to WT CMF, although it did not reach statistical significance (Fig. 3C).

On the other hand, feeding electrons directly into complex IV (CIV) by providing TMPD/ascorbate after adding AA to block electron flow from complex III (CIII) resulted in similar oxygen consumption for all cell lines (Fig. 3B), indicating that CIV activity is similar in cells containing or lacking functional XPC. Indeed, we could not detect any changes in CIV OCR in corrXP-C cells as well. This was confirmed by measuring cytochrome oxidase (COX) activity in cell extracts, where no differences in COX activity between WT and unXP-C were detected (Suppl. Fig. S3).

Together, these data show that mitochondria from XP-C cells display an imbalanced electron transport chain, with a significant decrease in CI activity, an increase in CII activity and no change in CIV, suggesting that these cells have deregulated expression and/or assembly of the OXPHOS complexes. Accordingly, mRNA expression of CII subunits, SDHA and SDHB, were significantly higher in both transformed (unXP-C) and primary (XP17VI) cells when compared to their WT controls (MRC-5 and AS405, respectively) (Fig. 3D). Moreover, SDHB protein level was also increased in unXP-C compared to corrXP-C (Fig. 3E). It is noteworthy that CII is the only OXPHOS complex that is exclusively nuclear-encoded, while CI is the OXPHOS complex with the largest number of mtDNA-encoded subunits.

### Increased mitochondrial H_2_O_2_ production is associated with decreased GPx activity in XP-C cells

**Figure 4:**
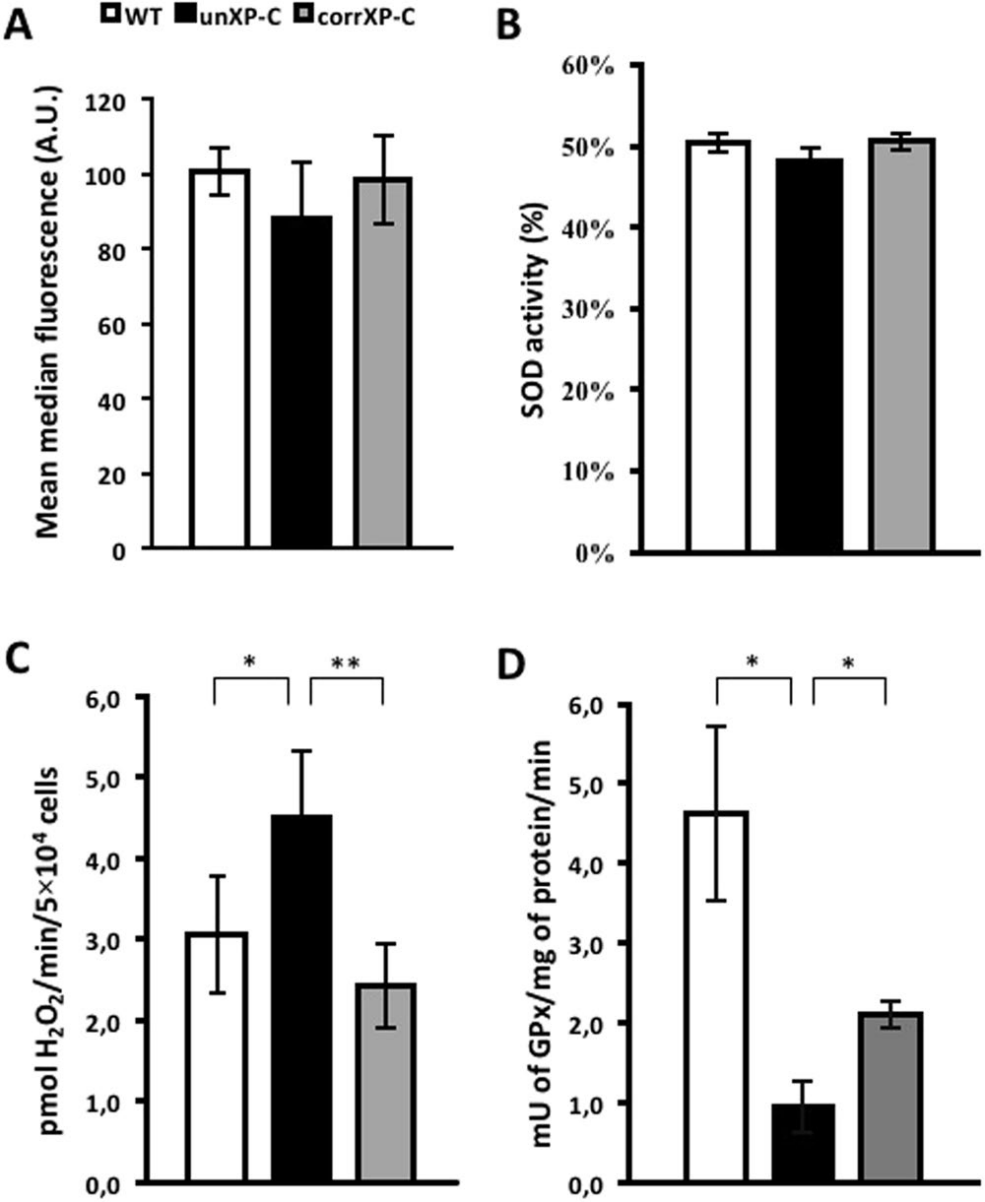
Redox imbalance in XP-C cells due to mitochondrial ROS production. (**A**) Superoxide generation was measured by flow cytometry using 1 × 10^5^ cells incubated with 5 µM Antimycin A, 5 µM DPI and 5 µM MitoSOX^®^. (**B**) H_2_O_2_ production was followed spectrofluorometrically with 5 µM Amplex UltraRed^®^, in presence of 5 µM Antimycin A, 5 µM DPI. Rate of H_2_O_2_ production calculated after calibration performed with known H_2_O_2_ concentrations. (**C**) SOD activity was measured using the Abcam kit, following the manufacturer’s instructions. (**D**) GPx activity was measured using 100 µg of total cellular protein. Oxidation of NADPH to NADP+ was monitored at 340 nm on a temperature-controlled spectrophotometer, at 25 °C. The results presented are the mean ± SD of n = 3 for superoxide production, n = 4 for H_2_O_2_ production, n = 3 for SOD and n = 3 for GPx activity. *p < 0.05 and **p < 0.01.

In most aerobic cell types, mitochondria represent a quantitatively important ROS generation site^25^. Because we found impaired CI activity together with increased mtDNA damage and deletion, we measured O_2_^•−^ and hydrogen peroxide (H_2_O_2_) production in WT, unXP-C and corrXP-C cells using fluorescent probes and assay conditions that allowed us to discriminate mitochondrial vs. non-mitochondria generation. Antimycin A-induced (AA) mitochondrial O_2_^•−^ production, measured in presence of the NOX inhibitor diphenyliodonium (DPI), was not altered in any of the three cell lines (Fig. 4A). As expected, mild-treatment with the mitochondrial uncoupler CCCP decreased MitoSOX^TM^ signal in all cell lines (not shown). On the other hand, H_2_O_2_ generation was significantly higher in unXP-C cells, from 5.7 ± 1.9 pmols/min in WT cells to 9.0 ± 1.1 pmols/min in the unXP-C cells, even in the presence of DPI and AA (Fig. 4B). The increased H_2_O_2_ generation was dependent on XPC deficiency, as corrXP-C cells produced similar levels of H_2_O_2_ as WT cells (Fig. 4B).

The increased H_2_O_2_ production in XP-C cells was not directly linked to a higher O_2_^•−^ dismutation rate, as total SOD activity was similar in all cell lines (Fig. 4C). However, GPx activity inversely correlated to H_2_O_2_ production, with a significant decrease in GPx in unXP-C cells, which is partially corrected after XPC expression in corrXP-C cells (Fig. 4D). Since SOD activity was unchanged, the elevated H_2_O_2_ level is likely the result of a decreased rate of H_2_O_2_ removal by GPx and other detoxifying systems, contributing to a possible role of GPx for an unbalanced mitochondrial redox status (Fig. 4D). Together, these results indicate that unXP-C cells are under redox imbalance, likely due to mitochondrially-generated H_2_O_2_.

### XP-C cells accumulate irreversible mtDNA damage

**Figure 5:**
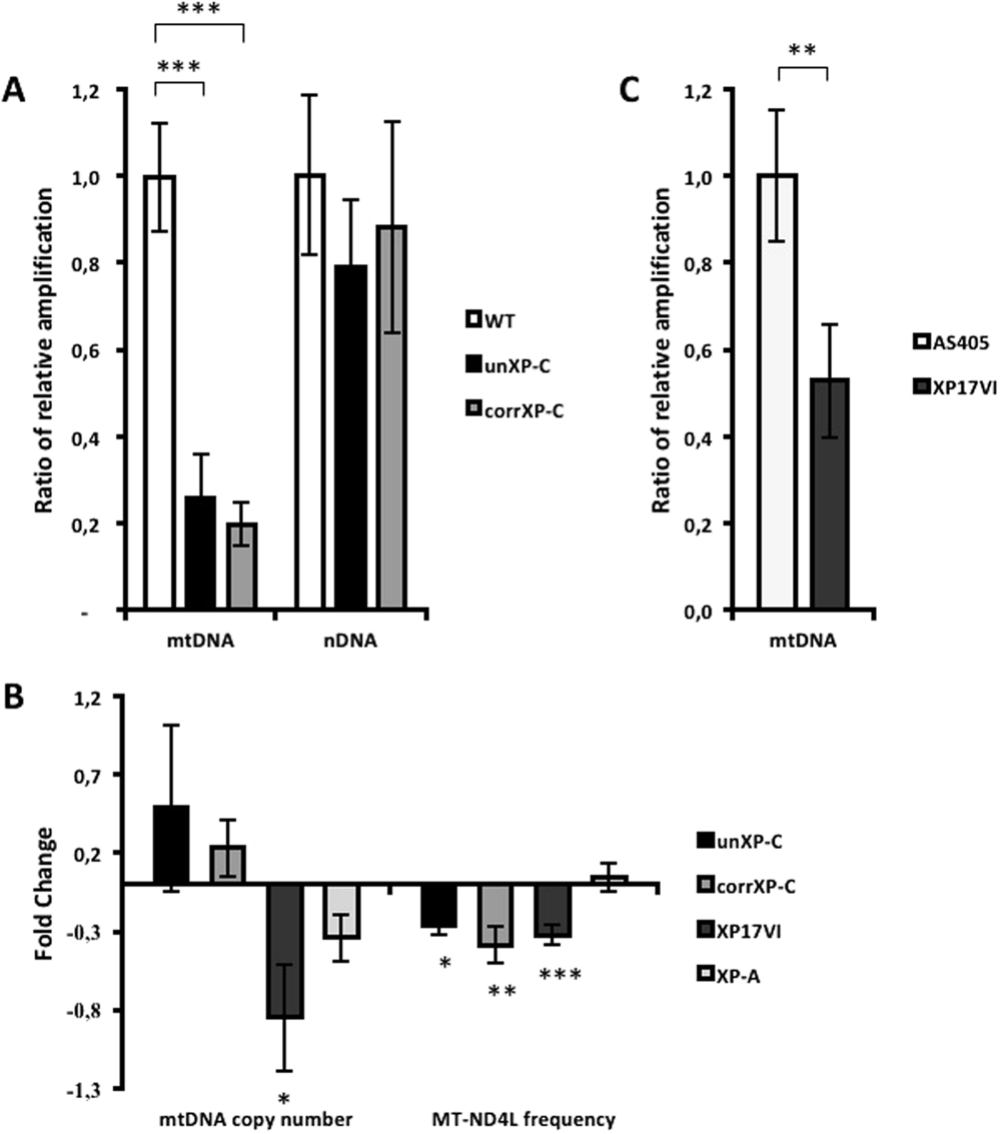
Accumulation of DNA damage in mtDNA in XP-C cells. DNA damage was measured by XL-PCR amplification of a 16.3 kbp fragment of the mtDNA (**A**, **left**, and **C**) and a 10.4 kbp fragment of the *HPRT* gene, for the nuclear genome (**A**, **right**). Data is presented as ratio of bands intensity of long fragment over small fragment loading control (*MT-ND1* and *HPRT* to mt and nDNA, respectively) relative to WT. The data represent mean ± SD in transformed and primary cells (n = 3). (**B**) MtDNA copy number and *MT-ND4L* frequency was assessed as the relative amplification of *MT-ND1* (mtDNA gene) and *HPRT* (nDNA gene), and of *MT-ND4L* gene (frequently deleted) vs. *MT-ND1* gene (rarely deleted), as described. WT and AS405 gene expression fold change are implicit and were set to 0.0 (zero) as control reference. The data represent mean ± SD in transformed (WT, unXP-C, corrXP-C and XP-A, n = 4) and primary (AS405 and XP17VI, n = 4 and n = 6, respectively) cells *p < 0.05, **p < 0.01 and ***p < 0.001.

mtDNA is physically associated with the inner mitochondrial membrane, and thus a primary target of mitochondrial-generated ROS^26^. Mitochondrial CI is the ETC complex with the largest number of subunits encoded by mtDNA, with 7 of the 13 mtDNA encoded polypeptides being components of CI (*MT-ND1*, *2*, *3*, *4L*, *4*, *5* and *6*). Because CI is impaired in XP-C cells and XP-C mitochondria are under a redox imbalance, we asked whether mtDNA is preferentially damaged and/or deleted. Using a long-extension PCR assay, which measures the inhibition of amplification of a large fragment (16 kb for the mtDNA and 10 kb for nDNA) by polymerase blockage at DNA lesions in the template, we found that the mtDNA is significantly more damaged in unXP-C cells when compared to WT normal cells, displaying an efficiency of amplification of only 20% that of the normal cells (Fig. 5A, left bars). On the other hand, in nuclear DNA, we detected only a small decrease in amplification efficiency in the unXP-C cells, which was not statistically significant (Fig. 5A, right bars). Together, these results indicate that, in cells lacking XPC, even under unstressed conditions, mtDNA accumulates significant amounts of DNA damage, while nuclear DNA is only marginally damaged.

Moreover, mitochondria from unXP-C cells had more deleted mtDNA molecules than mitochondria from normal cells, as the ratio of amplification of a region of the mitochondrial genome which is frequently deleted in humans, at the *MT-ND4L* gene, to a region which is seldom deleted, the *MT-ND1* gene^27^, was significantly lower in the unXP-C than in WT (Fig. 5B, right bars). It is noteworthy that this observed alteration was not due to decreased mtDNA copy number since we could not detect changes in the relative amplification of mtDNA (*MT-ND1*) *vs.* nDNA (*HPRT*) between unXP-C relative to WT cells (Fig. 5B, left bars).

To make sure that the accumulation of mtDNA damage was indeed a result of absence of XPC, we used the unrelated cell line XP17VI, a XP-C derived primary fibroblast, and AS405, a normal primary fibroblast cell line. XP17VI cells also showed accumulation of mtDNA damage (Fig. 5C) and deletions when compared to the normal individual, AS405, since the ratio of *MT-ND4L*/*MT-ND1* amplification was significantly lower (Fig. 5B, right bars). In this cell line, however, we detected a significant decrease in mtDNA copy numbers when compared to its control (AS405, Fig. 5B left bars).

However, to our surprise, mtDNA damage and *MT-ND4L* frequency were unchanged in corrXP-C cells compared to isogenic unXP-C cells (Fig. 5A and B). These results indicate that mtDNA integrity is irreversibly compromised in the XPC-deficient cells and cannot be reversed by the expression of wild type XPC in this background. Furthermore, this phenomenon seems to be specifically related to XPC deficiency, since no alteration in *MT-ND4L* frequency in XP-A relative to WT cell line was detected (Fig. 5B, right).

### XPC does not localize to mitochondria in unstressed cells

**Figure 6:**
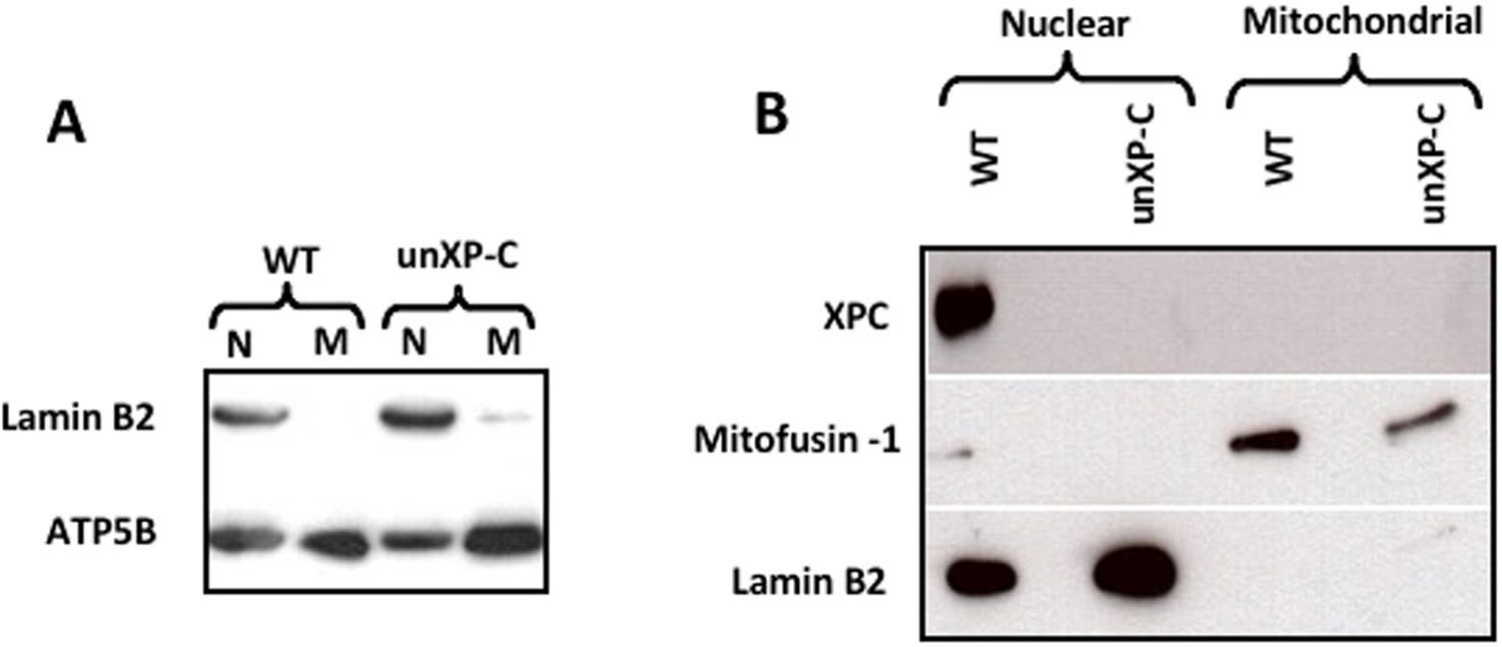
Expression and localization of the XPC protein in the cell. (**A**) Purity of mitochondrial extracts was checked by western blotting, using 10 µg of nuclear (N) or mitochondrial (M) fractions from MRC-5 (WT) or XP4PA (unXP-C) per lane. Lamin B2 was used as nuclear marker and ATP5B as mitochondrial. Mitochondrial localization of XPC was analyzed by western blotting. (**B**) Western blotting of mitochondrial and nuclear fractions used 40 μg of protein and anti-XPC, anti-Mfn1 as mitochondrial marker and anti-Lamin B2 as nuclear marker.

Since the XPC protein has been directly linked to repair of oxidized DNA bases via a physical interaction with OGG1^10^, and we detected significant accumulation of mtDNA damage in the XP-C cells, we investigated whether XPC localized in mitochondria. For that, we isolated highly purified mitochondria from WT and unXP-C cells, as demonstrated by the absence of Lamin B2 in the mitochondrial extracts (Fig. 6A). Those extracts were then used to check for XPC presence by western blots. As shown in Fig. 6B, XPC was detected in nuclear but not in mitochondrial extracts from WT cells, while no XPC signal was detected in either nuclear or mitochondrial extracts of unXP-C, since the transcript is unstable in these cells^28^. The purity of the extracts was again confirmed by probing for mitofusin 1 (mitochondrial protein) and Lamin B2 (nuclear protein). The results demonstrate that, under normal growth conditions, the XPC protein is mainly localized in the nucleus and does not localize in mitochondria. These observations suggest that the mitochondrial dysfunction detected in unXP-C cells is due to a role of XPC that is independent of its localization inside the organelle.

### NRF1 and SIRT3 are upregulated in XP-C cells

**Figure 7:**
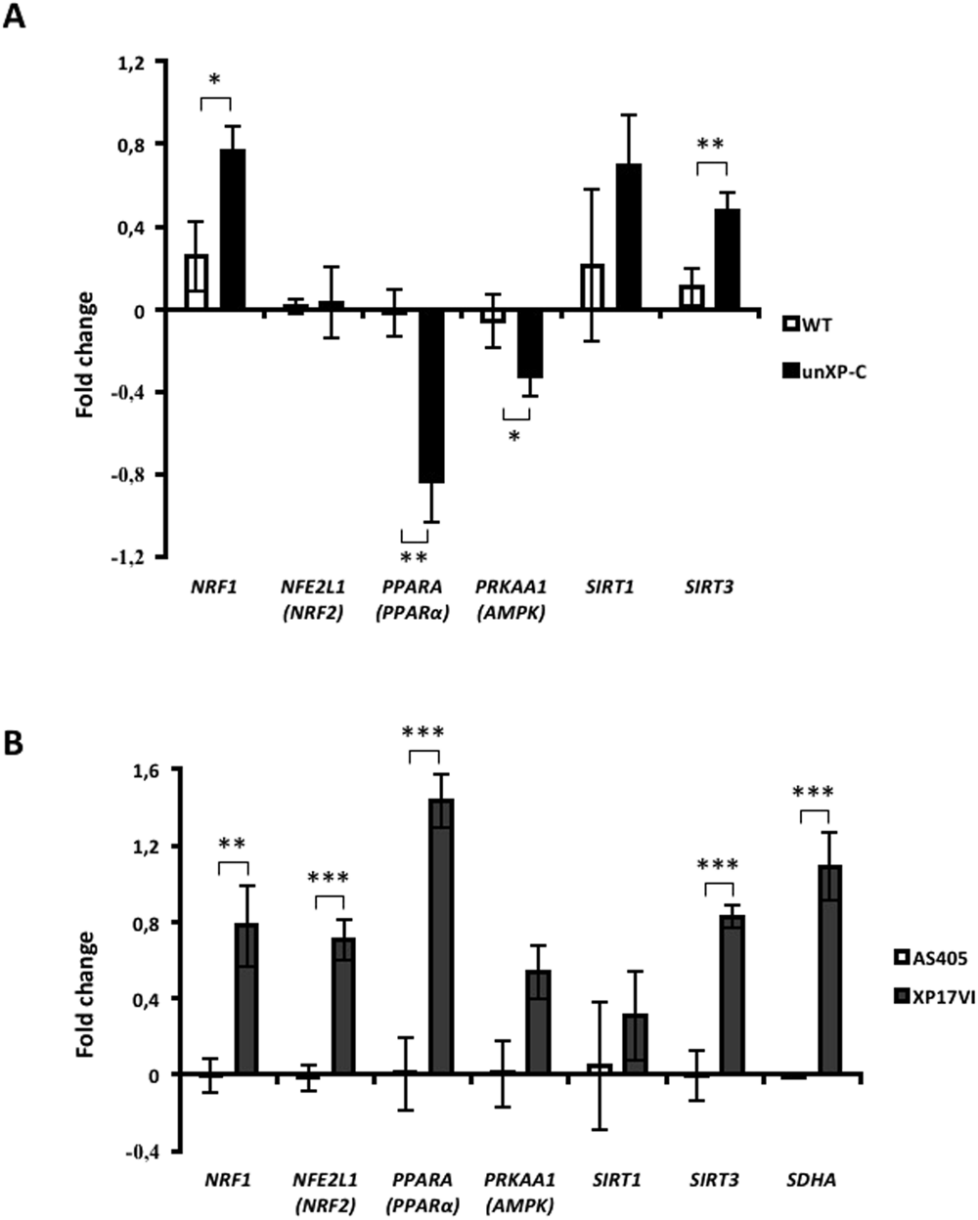
Expression analysis of mitochondrial genes by RT-qPCR. Fold-change in mRNA expression of XP4PA (unXP-C) relative to the wild type cell line (MRC-5). ACTB was set as reference gene for all genes analyzed. WT gene expression fold change and were set to 0.0 (zero) as control reference. The data represent mean ± SD of 4 independent experiments. *p < 0.05, **p < 0.01 and ***p < 0.001.

Because ETC subunits and other redox-related mitochondrial proteins are regulated through transcriptional programs and post-translational modifications, we checked expression levels of critical genes involved in mitochondrial biogenesis and modulation of mitochondrial metabolism. Two genes, nuclear respiratory factor 1 (NRF1) and sirtuin 3 (SIRT3), were found to be upregulated in transformed unXP-C and primary XP17VI comparing to their WT counterpart (Fig. 7A for transformed, and 7B for primary cells). These results indicate that lack of XPC leads to upregulation of transcription factor NRF1 and mitochondrial deacetylase SIRT3 in both transformed and untransformed fibroblasts.

### XP-C cells are more sensitive to mitochondrially-induced oxidative stress

Although total mitochondrial respiration was not impaired in XP-C cells, we hypothesized that the unbalanced redox status and mitochondrial ETC adaptive shift could sensitize mitochondria from cells lacking XPC to stress. To test that, we used the CIII inhibitor AA, which efficiently traps electron flow through the ETC. Consequently, there is an increase in one electron reduction of O_2_ generating O_2_^•−^ and increased mitochondrial oxidative stress^29^. We also challenged cells with MB-induced singlet oxygen (^1^O_2_) using a treatment protocol that excludes MB from the nucleus. Because ^1^O_2_ is able to generate 8-oxoGua via endoperoxide formation across C4-C5 and N7-C8 double bounds of guanine^30^, this treatment induces oxidative DNA damage accumulation exclusively in the mtDNA.

**Figure 8:**
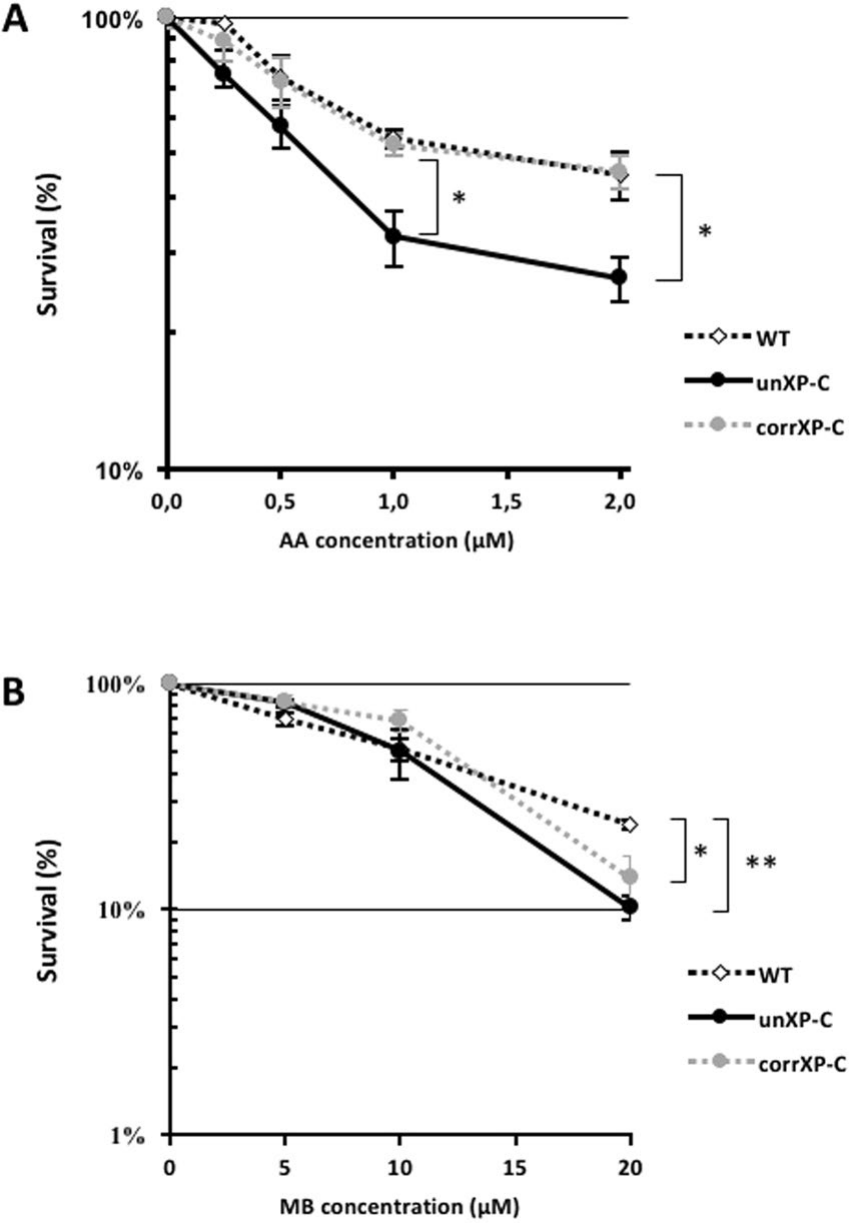
Absence of XPC sensitizes cells to AA and MB-induced cell death. Survival rates of cells treated with (**A**) antimycin A for 4 h or with (**B**) methylene blue subjected to photoactivation during 30 min. After treatments, individual cells were seeded at 500 cells/plate and allowed to grow to visible colonies. Survival was calculated relative to the plating efficiency of untreated cells. Values represent the mean ± s.e.m. of 4 independent experiments. *p < 0.05 and **p < 0.01.

As seen in Fig. 8A, the unXP-C cell line was more sensitive than WT at the two highest concentrations of AA (WT = 54.0% ± 2.4 *vs.* unXP-C = 32.7% ± 4.6 and WT = 44.8% ± 5.3 *vs*. unXP-C = 26.5% ± 2.9 for AA 1.0 and 2.0 μM, respectively) (*p* < 0.05). Although not statistically significant, cytotoxicity at 0.25 and 0.50 μM of AA was more pronounced in the unXP-C cells as well. These cells were also more sensitive to photoactivated MB at the highest concentration, 20 μM (WT = 23.9% ± 1.2; unXP-C = 10.2% ± 1.2) (*p* < 0.01) (Fig. 8B). The presence of XPC protein in corrXP-C cells was sufficient to protect cells from the toxic effects of mitochondrial oxidative stress induced by AA at all concentrations tested, suggesting that the ETC shift and redox imbalance observed in the absence of XP-C, which is reverted in the corrected cell line, may contribute to the increased sensitivity to AA. However, XPC expression in the corrXP-C cell line failed to revert the cytotoxicity induced by MB + light (Fig. 8B). Taken together, these results indicate that the lack of XPC protein sensitizes cells against mitochondrial respiratory chain blockage, but not singlet oxygen toxicity.

## Discussion

Several recent evidences have indicated that DNA repair deficiency is mechanistically linked to mitochondrial, and hence metabolic, dysfunction. Most notably, in XP-A^19^, AT^16–18^ and CS cells and animal models^12, 13, 31^, there is significant redox imbalance and increased metabolic rate due to deficient mitophagy. A link between defective DNA repair, tumorigenesis and mitochondrial dysfunction is also strengthened by results in an AT mouse model, where preventing mitochondrial dysfunction delayed tumor development^17^. In the CS and AT, however, a fraction of the proteins mutated in these syndromes, CSA and CSB in case of CS, and ATM in AT, localize to mitochondria and their absence partially explain mitochondrial symptoms^32^. These effects could be, at least in part, attributed to a direct role of the proteins in maintaining mtDNA integrity^18^. In the case of XP proteins (A-G), however, mitochondrial localization has been reported, so far, only for XPD. In fact, we show here that XPC cannot be detected in mitochondrial extracts, at least in unstressed cells (Fig. 6). Nonetheless, some XP symptoms, notably the neurodegeneration phenotype, overlap with classical mitochondrial diseases^33^ and impaired oxidative DNA damage repair has been implicated in XP etiology^21^. Moreover, mitochondrial dysfunction and accumulation of mtDNA damage are strongly associated with tumor development^21^, a hallmark feature of XP.

Recently, Rezvani and coworkers^22, 23^ demonstrated that XPC silencing in keratinocytes led to NOX-1 activation and cytosolic H_2_O_2_ production, suggesting that mitochondrial dysfunction is secondary to these events. However, we show here that mitochondria are a primary source of H_2_O_2_ production in XP-C cells, even in presence of a NOX-1 inhibitor (Fig. 4). This increased mitochondrial H_2_O_2_ production is accompanied by decreased GPx activity, indicating an intracellular redox imbalance. In addition, the mtDNA is significantly more damaged than the nuclear DNA in these cells, even in cells not exposed to any DNA damaging agent (Fig. 5). Using a long-extension PCR method, which detects the presence of any polymerase-blocking lesion, we detected a significant accumulation of lesions in mtDNA and only a small, non-significant level of lesions in the nDNA from XP-C cells. Because the mtDNA is physically associated with the mitochondrial inner membrane, this result also supports the hypothesis that mitochondria are a primary source of ROS in XP-C cells and that mtDNA is a primary target for damage accumulation. In contrast, the low levels of nDNA lesions suggest that, at least under unstressed conditions, the nDNA repair defect does not contribute significantly to the cellular phenotypes observed in the XP-C cells. Moreover, we detected higher levels of mtDNA deletions both in transformed and in primary XP-C cells. MtDNA deletions are suggested to result from incomplete repair events^34^; thus, this observation also supports the idea that the mtDNA is constantly being damaged by mitochondrially-generated H_2_O_2_, like hydroxyl radical. This is in agreement with the observation that XPC correction does not revert mtDNA damage, since the mtDNA pool in the XP-C cells has already been irreversibly damaged due to the exposure to H_2_O_2_ before the cells were corrected. Thus, we can speculate that lesions accumulated in mtDNA and mutations that arise from them could be used as biomarkers of cell exposure to chronic imbalanced redox status, even though the redox balance has been restored.

Several authors propose that accumulation of mutations in mtDNA plays an important role in the tumorigenic process, including skin cancer associated with exposure to UV^35^, the leading type of cancer observed in XP-C patients. On the other hand, recent results from large-scale sequencing of individual mitochondrial genomes from colorectal cancer patients showed lower mtDNA mutational burdens in cancer samples compared to normal samples^36^. These authors suggest that a shift from aerobic to glycolytic metabolism during tumor progression decreases ROS generation, thus “protecting” the mtDNA from oxidative damage induced mutagenesis. However, they do not rule out the possibility that primary mtDNA mutations contribute to the metabolic changes observed during the process.

Since XPC does not localize to mitochondria in non-stressed conditions, it is unlikely that mtDNA damage accumulation results from impaired repair, making it, more likely, an event secondary to increased mitochondrial ROS generation. The relevant question, thus, becomes how the lack of XPC leads to increased mitochondrial ROS generation? In this regard, we detected a pronounced decrease in CI-linked oxygen consumption and activity, associated with an increase in CII-linked respiration. This CI/CII shift cannot be directly explained by expression levels, since subunits of both CI and CII were found to be upregulated in XP-C cells (Figs S2A and 3D,E). Surprisingly, a recent study conducted Hosseini and coworkers found the opposite effect in *Xpc null* mouse keratinocytes. However, in that model downregulation of NADH:ubiquinone oxireductase, succinate dehydrogenase and cytochrome c oxidase was linked to premature skin aging, not to cellular transformation^37^. CI is believed to produce O_2_^•−^ at two sites, the flavin mononucleotide (FMN) and the ubiquinone (UQ) binding sites, thence CI and III are known sources of ROS formation^29^. In isolated mitochondria, a reverse electron flow from reduced UQ to CI has been detected^20^. On the other hand, succinate dehydrogenase complex is more likely to prevent ROS formation due to the intrinsic arrangement of its redox centers^38^. The assembly of CI into the mature holoenzyme involves the coordinated expression of the two genomes, plus a large group of proteins known as “assembly factors”^39^. Mutations in genes of both structural and/or catalytic proteins, as in mounting factors, result in loss of activity of the complex. Even when the catalytic activity is preserved, as in the case of Leber’s hereditary optic neuropathy (LHON) caused by mutations in three mitochondrial genes of CI, there is an increase of sensitivity to inhibitors of CI respiration^40^, and associated H_2_O_2_ generation.

Although ROS have been classically linked to cellular decay, recent attention has been given to their functions in cell signaling. Consistently, H_2_O_2_ lower reactivity (when compared to radical species, like ^•^OH) added to its relatively high diffusion rate prompted this species to be considered the main ROS in cell signaling^41^. Accordingly, Piantadosi & Suliman demonstrated that low levels of oxidative stress trigger nuclear respiratory factor 1 (NRF1) phosphorylation and cytosol to nucleus translocation^42^. NRF1 is a major transcription factor that activates the expression of some key metabolic genes, regulating cellular growth and the expression of nuclear genes required for respiration, heme biosynthesis, and mitochondrial DNA transcription and replication. The same group demonstrated that NRF1 is recruited to two promoters of CII subunits, SDHA and SDHD^43^. Moreover, ChIP-seq analysis revealed not only SHDA, B and D as NRF1 target genes, but also from all five ETC complexes and some DNA repair pathways, more specifically BER and non-homologous end joining^44^. Indeed NRF1, SDHA and SDHB expression levels are increased in unXP-C cells and unrelated XP17VI primary fibroblasts compared to their WT counterpart (Figs 3D,E and 7).

In keratinocytes, Han and coworkers demonstrated a role of NRF1 linking redox homeostasis through promotion of enzymes involved in glutathione synthesis and UVB-induced DNA repair via GGR, more specifically, XPC expression. The expression of this DNA damage recognizing protein was significantly reduced upon NRF1 knockdown, as well as GSH/GSSG levels. Interestingly, treatment with cell permeable glutathione (GSH) was sufficient to restore XPC expression even in the absence of NRF1, demonstrating that redox homeostasis in vital to XPC transcription in keratinocytes^45^.

While CII is not classically linked to ROS generation, mutations in some subunits of this complex give rise to cancer-prone diseases^46^. CII dysfunction leads to succinate accumulation with subsequent prolyl hydroxylase inhibition allowing stabilization of HIF1α, which may act as an oncogene^47^. Furthermore, cellular studies expressing mutant SDHC (a subunit of CII) show that CII dysfunction gives rise to sub-lethal doses of ROS, promoting genomic instability and cell proliferation that favors tumorigenesis^48, 49^. More recently, a role for CII in ROS sensing and generation in apoptosis has also been proposed. CII displays two distinct enzymatic activities i) succinate dehydrogenase (SDH) and ii) succinate ubiquinone oxireductase (SQR)^50^. During the course of apoptosis, SDHA and B, which encompass the SDH activity, are dislodged from the membrane-anchored complex. SDH activity continues to operate removing electrons from succinate to FAD and Fe-S clusters in released SDHA/B, but apart from SDHC/D, the membrane-bond ubiquinone oxireductase site, O_2_ undergoes one-electron reduction producing O_2_^•−^, overflowing the cell with ROS culminating with apoptosis^50–52^. Thus, it is possible that under conditions in which CI and CII are unbalanced, like that seen in the XP-C cells, CII activity contributes to chronic H_2_O_2_ generation and genomic instability, and consequently to driving the tumorigenic process.

Together, these results indicate that XPC deficiency leads to mitochondrial-derived ROS formation as result of impaired CI activity, the major ETC complex encoded by mtDNA. Increased H_2_O_2_ production may trigger NRF1 leading to CII upregulation, as indicated by the increased succinate-linked OCR and CII + CIII activity. It is important to note that the CI/CII shift is completely dependent on the lack of XPC, as expression of the wild type protein reverts the phenotypes. As a consequence of this adaptive shift between CI and CII fueling preference, XP-C cells may counteract mitochondrially-generated ROS reducing the impact of imbalanced redox status, but at the cost of sensitivity to mitochondrial oxidative stress, considering that CII might have an important role as a sensor and an effector for apoptosis.

## Material and Methods

### Cell lines and culture

The human SV40-transformed fibroblast cell lines MRC-5, XP4PA, corrected XP4PA and XP12RO cell line (hereinafter WT, unXP-C, corrXP-C and XP-A), and the human primary fibroblasts cell lines AS405 (wilt type) and XP17VI (XP-C) were kindly provided by Prof. Dr. Carlos F. M. Menck, ICB, University of São Paulo. Both XP-C cell lines are homozygous for the prevalent mutation c.1643-1644delTG (1744–1745) in exon 9. All cell lines were maintained in DMEM/high glucose supplemented with 10% FBS, penicillin 100 IU/mL and streptomycin 100 μg/mL, at 37 °C in a humidified atmosphere with 5% CO_2_ (standard conditions). Cultures were routinely sub-cultured, by trypsinization, when reached up to 80% confluence.

### Determination of O_2_ consumption

O_2_ consumption rates (OCR) of WT, unXP-C and corrXP-C cells (2 × 10^6^ cells/mL) were measured using a Hansatech electrode (Hansatech Instruments Ltd.) and the high-resolution respirometry (HRR) O2k-core (Oroboros). Cells were permeabilized with 30 µM digitonin in 2 mL of standard reaction medium (125 mM sucrose, 65 mM KCl, 10 mM HEPES, 2 mM K_2_HPO_4_, 1 mM MgCl_2_, pH 7.2) containing 0.33 mM EGTA, 5 mM CI-linked substrate cocktail (3.4 mM malate, 1.86 mM α-ketoglutarate, 2.1 mM pyruvate, 2.1 mM glutamate). Sequential additions of 100 µM ADP, 4 µM CAT and 0, 5 µM CCCP were made to analyze respiratory states 2, 4 and uncoupled respiration, respectively. CII, III and IV OCR were also measured using HRR O2k-core with the same 2 mL standard reaction medium. Cells were permeabilized with 30 µM digitonin, with the following sequential additions: 5 mM succinate, 0.25 µM CCCP, 1 µM AA and 200 µM TMPD + 2 mM ascorbate. All analyses were made at 37 °C.

In order to evaluate more physiological respiratory parameters, OCR was measured in intact cells using XF24 Extracellular Flux Analyzer (Seahorse Bioscience). Approximately 4 × 10^4^ cells per well were seeded in XF24 V7 Cell Culture Microplates at standard culture conditions. After 12 h, DMEM was washed off with 1 mL of XF Assay Medium supplemented with 10 mM D-glucose and cells were incubated in humidified CO_2_-free incubator at 37 °C for 1 h.

Respiratory parameters were analyzed based in the manufacturer’s protocol for the Seahorse XF Cell Mito Stress Test Kit with slight modifications. Sequential additions of 1 µM oligomycin, 200 nM CCCP and 1 µM rotenone plus AA were made to analyze basal respiration, ATP production, proton leak, maximum respiratory capacity, spare respiratory capacity and non-mitochondrial respiration.

### DNA damage quantification by XL-PCR

DNA damage in the mitochondrial or nuclear DNA was estimated measuring PCR amplification efficiency of a long fragment of each genome (over 10 kilobases)^53^. DNA samples were isolated using the DNeasy Blood & Tissue Kit^®^ (Qiagen), according to the manufacturer’s instructions. Long-extension PCR (XL-PCR) reactions were performed as described^54^, with minor modifications. Cycling parameters were modified to include an annealing step of 30 s at 64 °C, and an increase in the denaturing step to 30 s.

DNA amplicons were submitted to electrophoretical separation in 0.8% agarose gel at 5 V/cm for 4 h in TAE buffer (40 mM Tris-acetate pH 7.8, 1 mM EDTA). Gels were stained in 2 mg/L ethidium bromide for 30 min and distained in deionized water for 10 min. Images were captured and analyzed by ImageJ (NIH). Equal DNA template loading was corrected by ΔCt of qPCR for mtDNA copy number, as described below. We chose to use qPCR instead gel electrophoresis densitometry of small mtDNA as control due to smaller variation between samples, although means were comparable (not shown).

### Determination of mtDNA copy number and deletion levels

DNA samples were isolated using DNeasy^®^ Blood & Tissue Kit (Qiagen) according to the manufacturer’s instructions. Real time quantitative PCR (qPCR) was performed using SYBR Green PCR Master Mix (Applied Biosystems) according to the manufacturer’s instructions. Data analysis was performed by ΔΔCt method and adjusted to fold-change. The following genes were analyzed by qPCR: *HPRT* for nDNA, *MT-ND1* for mtDNA copy number and *MT-ND4L* for mtDNA deletion. *MT-ND4L* was chosen as a deletion marker due to the high susceptibility of the genomic region in which this gene is located^27, 55^. The primer sequences are presented in Supplemental Table 1A.

### Mitochondrial isolation from cell cultures

Mitochondria were isolated using differential centrifugation and Percoll gradient, as described^56^.

### Western blotting

Nuclear and mitochondrial fractions were analyzed by Western blotting using standard techniques. Incubations with primary antibodies were performed in 1% non-fat dry milk in TBS-T, either 1–2 h at room temperature or overnight at 4 °C, with the following antibodies: anti-actin, N-terminal (1:2000; rabbit A2103, Sigma), anti-Lamin B2 (X223) (1:1000; mouse sc-56147, Santa Cruz Biotechnology), anti-ATPB antibody [3D5] (1:1000; mouse ab-14739, Abcam), anti-Mfn1 (H-65) (1:500; rabbit sc-50330, Santa Cruz Biotechnology), anti-XPC (1:400; rabbit ab21078 or 1:250; [3.26] mouse ab6264, Abcam), anti-NDUFS4 (2C7CD4AG3) (1:X; mouse ab87399, Abcam) and Complex II Western Blot Antibody Cocktail (1:X; mouse MS202, MitoSciences). After washing with TBS-T, appropriate secondary antibodies linked to horseradish peroxidase were applied at 1:5000 (bovine anti-goat, goat anti-rabbit or goat anti-mouse, Santa Cruz Biotechnology) in 1% milk in TBS-T. Membranes were then washed repeatedly with TBS-T and visualized using Amersham ECL Plus (GE Healthcare).

### Mitochondrial Superoxide Production

For quantification of mitochondrial superoxide generation, approximately 3 × 10^5^ cells were seeded 48 h prior to the experiment in 60 cm^2^ dishes (TPP). WT, unXP-C and corrXP-C cells were washed in pre-warmed PBS, trypsinized and centrifuged for 5 min at 300 *g* at 37 °C. The cell pellets were suspended in 0.22 μm filtered Hank’s Balanced Salt Solution (HBSS) supplemented with D-glucose 1 g/L in an approximate final concentration of 1 × 10^6^ cells/mL. Cells were incubated for 30 min at 37 °C with: (i) 5 µM MitoSOX (Molecular Probes); (ii) 5 µM MitoSOX, 5 µM antimycin A (AA) (CIII inhibitor) and 5 µM DPI (diphenylene iodonium – to inhibit NOX-generated superoxide formation); and (iii) 5 µM MitoSOX and 500 nM CCCP (for uncoupling). Fluorescence intensity was analyzed by flow cytometry, in the FL-2 channel as described^56^. Results shown are relative to treatment ii) with MitoSOX + AA + DPI. TMRM controls were also performed (data not shown).

### H_2_O_2_ Production

Hydrogen peroxide production in WT, unXP-C and corrXP-C cells was determined fluorimetrically following the oxidation of Amplex UltraRed (Molecular Probes) to the highly fluorescent compound, resorufin^57^. For that, cells were incubated in HBSS containing 5 µM Amplex UltraRed and 0.1 U/mL horseradish peroxidase (HRP). Fluorescence was monitored over time in SpectraMax 190 reader (Molecular Devices) in 96-well plate format using excitation and emission wavelengths of 530 and 590 nm respectively, with low sensitivity. Under these conditions, a linear increase in fluorescence indicates an increase in H_2_O_2_ formation. Calibration curves were made with known concentrations of H_2_O_2_.

### Measurements of enzyme activities

Glutathione peroxidase (GPx) activity was determined by measuring the rate of formation of oxidized glutathione form reduced GSH in the presence of H_2_O_2_, detected by the change in absorbance at 340 nm due to NADPH oxidation, as previously described^58^.

Superoxide dismutase (SOD) activity was measured using the SOD activity colorimetric assay kit^®^ (Abcam) according to the manufacturer’s protocol, in a microplate at 37 °C at 450 nm.

CI + III and CII + III activities was measured as previously described^59, 60^ with slight modifications. Reactions were adjusted to fit in 96-well microplate platform. NADH cytochrome c oxidoreductase and succinate cytochrome c reductase activities were acquired in SoftMax Pro (Molecular Devices) and expressed as the rate of reduced cytochrome c formation per min per mg of protein.

Cytochrome c oxidase activity was measured using a protocol based on a commercial kit (Sigma-Aldrich), which follows the decrease in absorbance at 550 nm due to the oxidation of reduced cytochrome c. Enzyme activity was calculated using the difference in reduced and oxidized cytochrome c molar absorptivity coefficients of 21.84 mM^−1^ × cm^−1^.

Enzyme activities are expressed relative to the total protein content, determined by Bradford (1976), using bovine gamma globulin (Bio-Rad) as standard.

### Clonogenic assay

Clonogenic cell survival is regarded as gold standard test to detect cellular toxicity^’^. Briefly, 1.0 × 10^5^ cells were seeded in 6-well plate (TPP) and incubated for 24 h, at standard conditions. For MB treatment, cells were rinsed with PBS and incubated for 1 h with MB concentrations of 5.0, 10.0 and 20.0 μM diluted in DMEM without FBS. All further steps were carried out in a dark room aided with blue lamp. At the end of the incubation, cells were washed twice with PBS and incorporated MB was photoactivated with a 634 nm LED lamp for 30 min. Cells were then trypsinized, counted and approximately one thousand cells were seeded in 60 mm Petri dishes (Nunc, Thermo-Scientific) in triplicate. Cells were incubated in standard conditions for 8 days. For treatment with AA, 500 cells were plated in 6-well plate (TPP) in triplicate and incubated for 24 h in standard conditions. Cells were washed twice with PBS and incubated with AA at 0.25, 0.50, 1.0 and 2.0 μM in DMEM without FBS for 4 h. After that, AA was washed out with PBS and cells were incubated in complete medium in standard conditions for 6 to 7 days. Colonies were washed in ice-cold PBS, ethanol-fixed and stained with 1% (w/v) crystal violet solution. Survival rate was calculated as the ratio of number of colonies in treated over non-treated conditions.

### Analysis of gene expression

Total RNA was extracted using RNeasy Micro Kit (Qiagen), following the manufacturer’s instructions. The expression levels of *MT-ND1*, *MT-ND4L*, *NDUFS1*, *NRF1*, *NFE2L1* (NRF2), *PPARA* (PPARα), *PRKAA* (AMPK), *SIRT1*, *SIRT3*, *SDHA* and *SHDB* mRNA were measured by quantitative RT-qPCR assays, with primers targeting the mRNAs expressed by these genes, and β-actin (*ACTB*) as the loading control. Three other housekeeping genes were compared to *ACTB* expression (*TUBB*, *TBP* and *HPRT*) none of which showed significant difference (not shown). Total RNA extracted from WT, unXP-C, corrXP-C, AS405 and XP17VI cells was used to generate cDNA with the High Capacity cDNA Reverse Transcription kit (Applied Biosystems). Quantitative RT-PCR reactions were performed using the Power SYBR Green PCR Master Mix (Applied Biosystems), according to directions, using 100 ng template and 5 pmol of each primer. Cycling, reading and data analyses were performed as described above. The primer sequences are presented in Supplemental Table S2.

### Statistical analyses

Results are shown as mean ± standard deviation (SD) or standard error of the mean (s.e.m.), or as representative traces of at least 3 independent experiments. The number of repeats is stated in the figure legends. ANOVA with Tukey regression was used in WT, unXP-C and corrXP-C comparison, while Student’s *t* test was used to assess differences between two groups, considering significant *p* < *0.05*.

## Electronic supplementary material

Supplementary Information
