# Supplementary material for: Lack of XPC leads to a shift between respiratory complexes I and II but sensitizes cells to mitochondrial stress
Source: Sci Rep. 2017 Mar 13;7:155. doi: 10.1038/s41598-017-00130-x (PMC5427820; doi:10.1038/s41598-017-00130-x)
Supplement: Supplementary file 1 — Supplementary Information [file 41598_2017_130_MOESM1_ESM.pdf]

Lack of XPC leads to a shift between respiratory complexes I and II but sensitizes cells to mitochondrial stress

Mateus P. Mori[a,*], Rute A. P. Costa[b,*], Daniela T. Soltys[a], Thiago de S. Freire[a], Franco A. Rossato[b], Ignácio Amigo[a], Alicia J. Kowaltowski[a], Aníbal E. Vercesi[b], Nadja C. de Souza-Pinto[a,¶]

[a]Departamento de Bioquímica, Instituto de Química, Universidade de São Paulo (USP), São Paulo, SP, Brazil.
[b]Department of Clinical Pathology, School of Medical Sciences, Universidade Estadual de Campinas (UNICAMP), Campinas, SP, Brazil.

Supplemental Material

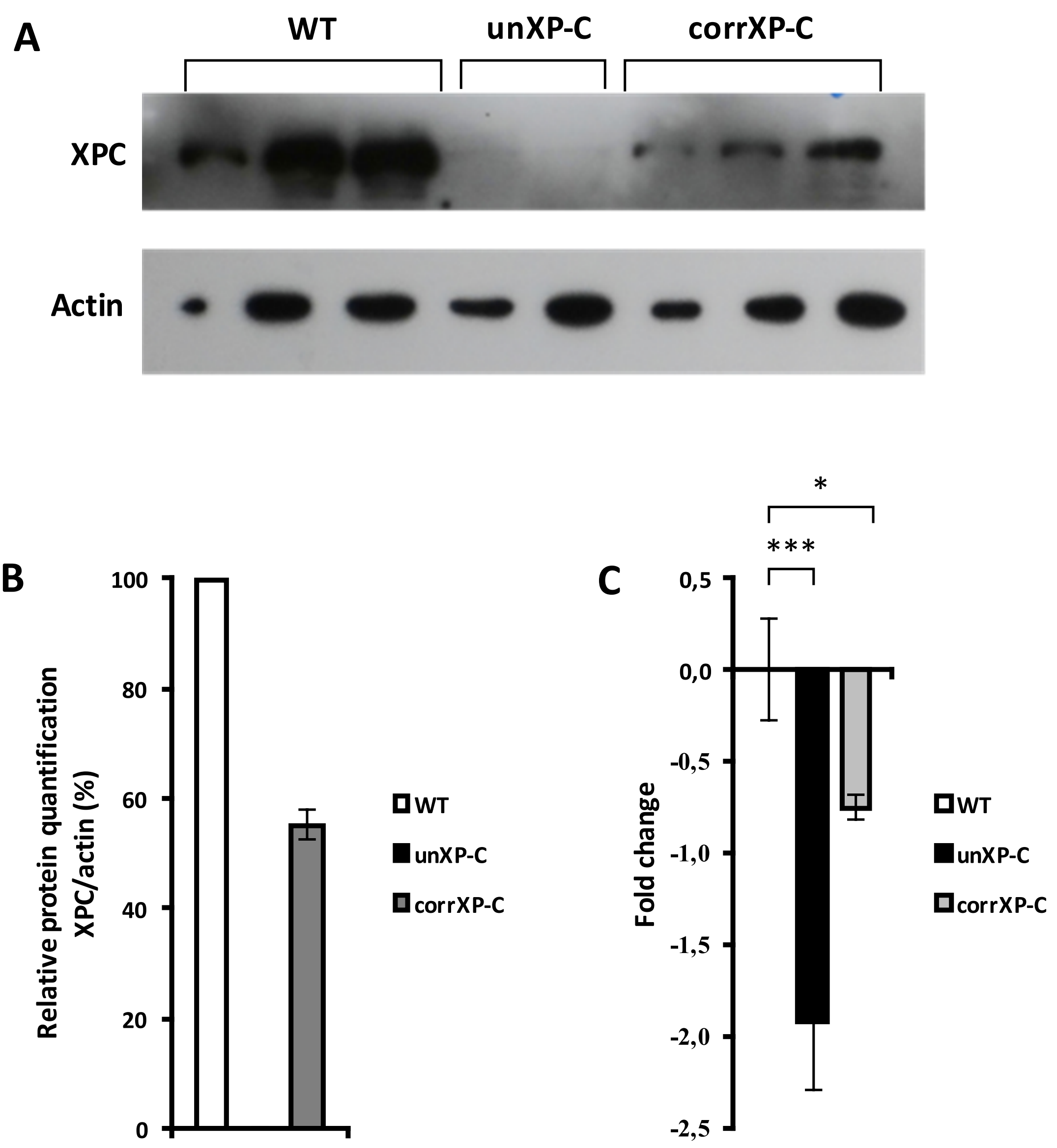

Figure S1: XPC protein and mRNA levels in immortalized fibroblasts cell lines. **A)** Western blot depicting XPC and β-actin protein levels in whole cell extracts. Images were captured and analyzed by ImageJ (NIH). **B)** XPC quantification relative to expression in WT cells, normalized to β-actin levels. No detectable levels of XPC protein were found in unXP-C cells; corrXP-C cells express approximately 57% of the levels detected in WT cells. **C)** Fold-change in mRNA expression of XPC gene in unXP-C and corrXP-C cells relative to WT cells measured by RT-qPCR. * $p < 0.05$ and *** $p<0.001$.

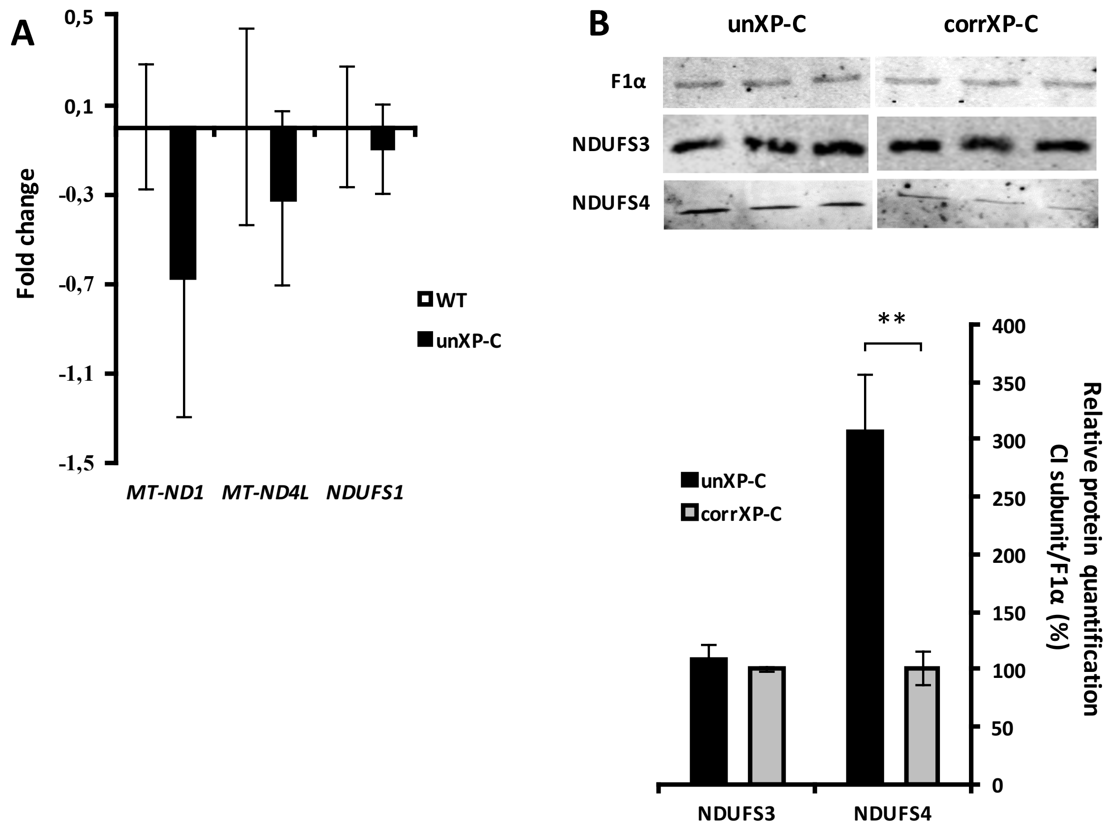

Figure S2: A) Expression analysis of *MT-ND1*, *MT-ND4L* and *NDUFS1* genes by RT-qPCR. Fold-change in mRNA expression in unXP-C relative to the WT cell line (MRC-5). ACTB was used as reference gene for all genes analyzed. The data represent mean ± SD of 3 independent experiments. **B)** Western blotting of CI subunits NDUFS3 and NDUFS4 and ATPase subunit F1α. CI subunits protein expression levels were normalized against F1α in unXP-C relative to corrXP-C cells. The data represent mean ± SD of 3 independent experiments. ** $p < 0.01$.

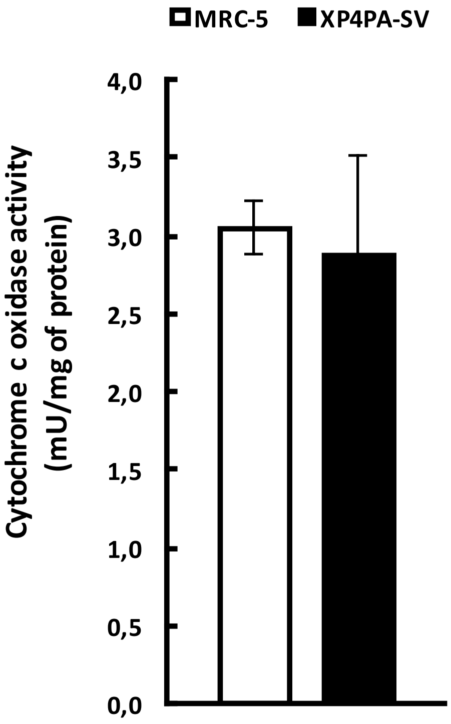

Figure S3: Cytochrome c oxidase activity. Cytochrome oxidase activity was measured in whole cell extracts, using 10 μg of total cellular protein in reactions containing 10 mM Tris-HCl (pH 7.0), 250 mM sucrose and 1 mM reduced cytochrome c. The oxidation of ferrocytochrome c to ferricytochrome c was monitored at 550 nm at 25°C. The results presented are mean ± SD of 4 independent experiments.

Gene	Gene name		Sequência	mRNA RefSeq
MT-ND1	mitochondrially encoded NADH 1 dehydrogenase	Fw	ACT ACG CAA AGG CCC CAA CG	NC_012920.1
		Rev	GAG CTA AGG TCG GGG CGG TG	
MT-ND4L	mitochondrially encoded NADH 4L dehydrogenase	Fw	ACC CCT CAA CAC CCA CTC CCT CTT	NC_0111137.1
		Rev	TAG GCC CAC CGC TGC TTC GC	
HPRT	hypoxanthine phosphoribosyltransferase	Fw	TGA CAT GTG CCG CCT GCG AG	NC_000023.11
		Rev	GTG GTC GCT TTC CGT GCC GA	
NCAPD2	non-SMC condensin I complex, subunit D2	Fw	ATG GTT GCC ACT GGG GAT CT	NC_000012
		Rev	TGC CAA AGC CTA GGG GAA GA	

Gene	Gene name		Sequência	mRNA RefSeq
XL-mtDNA	mitochondrially encoded NADH 1 dehydrogenase	Fw	TGA GGC CAA ATA TCA TTC TGA GGG GC	J01415
		Rev	TTT CAT CAT GCG GAG ATG TTG GAT GG	
XL-nuDNA	hypoxanthine phosphoribosyltransferase	Fw	TGG GAT TAC ACG TGT GAA CCA ACC	J00205
		Rev	GCT CTA CCC TCT CCT CTA CCG TCC	

Table S1: Genes and primers used in qPCR experiments. Top: primers used to investigate mtDNA copy number and deletion. *MT-ND1* was used as reference mtDNA gene and *MT-ND4L* was used as deletion marker[54,55]. *HPRT* and NCAPD2 were used as nDNA reference. Bottom: primers used in XL-PCR to detect mitochondrial and nuclear DNA polymerase blocking lesions.

Gene	Gene name		Sequence	mRNA RefSeq
MT-ND1	mitochondrially encoded NADH 1dehydrogenase	Fw	TAC AAC TAC GCA AAG GCC CC	NC_012920.1
		Rev	TGG TAG ATG TGG CGG GTT TT	
MT-ND4L	mitochondrially encoded NADH 4L dehydrogenase	Fw	TCG CTC ACA CCT CAT ATC CTC	NC_012920.1
		Rev	AGG CGG CAA AGA CTA GTA TGG	
NDUFS1	NADH:ubiquinone oxireductase core subunit 1	Fw	TTA GCA AAT CAC CCA TTG GAC TG	NM_001199981.1
		Rev	CCC CTC TAA AAA TCG GCT CCT A	
NRF1	nuclear respiratory factor 1	Fw	CGG AGC CTT GAT GTG GTA GG	NM_006251.5
		Rev	TCA TCC AGC CTT CCA TTC TTA CA	
NFE2L1 (NRF2)	nuclear factor, erythroid 2-like 1	Fw	TAC TCC CAG GTT GCC CAC A	NM_006164.4
		Rev	CAT CTA CAA ACG GGA ATG TCT GC	
PPARA (PPARα)	peroxisome proliferator-activated receptor alpha	Fw	GTC TCC CAG RGG AGC ATT GA	NM_005036.4
		Rev	ACC AGC TTG AGT CGA ATC GT	
PRKAA1 (AMPK)	protein kinase, AMP-activated alpha 1catalytic subunit	Fw	CGG AGC CTT GAT GTG GTA GG	NM_006251.5
		Rev	TCA TCC AGC CTT CCA TTC TTA CA	
SIRT1	sirtuin 1	Fw	TGG GTA CCG AGA TAA CCT TCT	NM_012238.4
		Rev	TGT TCG AGG ATC TGT GCC AA	
SIRT3	sirtuin 3	Fw	CAC AGT CTG CCA AAG ACC CT	NM_012239.5
		Rev	CAA TGT CGG GCT TCA CAA CG	
SDHA	succinate dehydrogenase complex, subunit A, flavoprotein (Fp)	Fw	CCT TTC TGA GGC AGG GTT TA	NM_004168.2
		Rev	AGA GCA GCA TTG ATT CCT CC	
SDHB	succinate dehydrogenase complex iron sulfur subunit	Fw	ACC TTC CGA AGA TCA TGC AGA	NM_003000.2
		Rev	GTG CAA GCT AGA GTG TTG CCT	
XPC	xeroderma pigmentosum, complementation group C	Fw	CAT CGT GGG AGC CAT CGT AAG	NM_004628.4
		Rev	CTC ACC ATC CGC TGC ACA TTT T	
TUBB	tubulin, beta class I	Fw	TGG ACT CTG TTC GCT CAG GT	NM_001293212.1
		Rev	TGC CTC CTT CCG TAC CAC AT	
TBP	TATA-box binding protein	Fw	CCA CTC ACA GAC TCT CAC AAC	NM_003194.4
		Rev	CTG CGG TAC AAT CCC AGA ACT	
HPRT	hypoxanthine phosphoribosyl transferase 1	Fw	GAA AAG GAC CCC ACG AAG TGT	NM_00194.2
		Rev	AGT CAA GGG CAT ATC CTA CAA CA	
ACTB	actin, beta	Fw	CTC TTC CAG CCT TCC TTC CT	NM_001101.3
		Rev	AGC ACT GTG TTG GCG TAC AG	

Table S2: Genes used to investigate expression of mitochondrial-related genes by RT-qPCR. *ACTB*, *TBP*, *TUBB* and *HPRT* were used as reference housekeeping genes, as indicated.
